# Interleukin-35 in autoimmune dermatoses: Current concepts

**DOI:** 10.1515/med-2022-0455

**Published:** 2022-03-21

**Authors:** Yuming Xie, Huilin Zhang, Junke Huang, Qing Zhang

**Affiliations:** Department of Dermatology, The Second Xiangya Hospital, Central South University, Hunan Key Laboratory of Medical Epigenomics, Changsha, Hunan 410011, China; Clinical Nursing Teaching and Research Section, The Second Xiangya Hospital, Central South University, Changsha, Hunan 410011, China; Department of Dermatology, The Second Xiangya Hospital, Central South University, Hunan Key Laboratory of Medical Epigenomics, #139 Renmin Middle Rd, Changsha, Hunan 410011, China

**Keywords:** dermatomyositis, interleukin-35, psoriasis, systemic lupus erythematosus, systemic sclerosis

## Abstract

Interleukin-35 (IL-35) is a lately observed cytokine and is part of the IL-12 cytokine family. IL-35 includes two subunits, p35 and Epstein-Barr virus-induced gene 3, and activates subsequent signaling pathways by binding to receptors to mediate signal transduction, thereby modulating the immunoregulatory functions of T cells, B cells, macrophages, and other immune cell types. Although there is currently limited research on the roles of IL-35 in human autoimmunity, many studies have demonstrated that IL-35 may mediate immunosuppression. Therefore, it plays an essential role in some autoimmune dermatoses, including systemic lupus erythematosus, psoriasis, systemic sclerosis, and dermatomyositis. We will introduce the structure and biological characteristics of IL-35 and summarize its effects on the occurrence and development of autoimmune dermatoses in this article. It is suggested that IL-35 is a possible target for therapy in the aforementioned diseases.

## 1 Introduction

As described recently, interleukin-35 (IL-35) is a new member of the IL-12 cytokine family, which also contains IL-12, IL-23, and IL-27 [1,2]. The members of the IL-12 cytokine family are all heterodimers made up of an α-subunit and a β-subunit. IL-12 consists of p40 and p35 subunits, IL-23 consists of p40 and p19, while IL-27 is made up of p28 and Epstein-Barr virus-induced gene 3 (Ebi3) [1,3,4]. It is reported that p35 and Ebi3 form IL-35 [1,2]. The two subunits of IL-35 can both regulate the immune activities independently, while p35 is the main subunit of IL-35 that plays an immunological role, and Ebi3 can form a heterodimer with p35 to enhance its function [5]. Mouse IL-35 can be expressed by non-stimulated CD4^+^Foxp3^+^ regulatory T cells (Tregs) [1]. However, human IL-35 is undetectable in non-stimulated Tregs but can be produced by Tregs after activation [6,7,8,9]. IL-35 is inducible in regulatory B cells (Bregs) [10], tolerogenic dendritic cells [11], and placental trophoblast cells [12].

IL-35 receptors are heterodimers or homodimers that are composed of IL-12Rβ2, gp130, or IL-27Rα, including two heterodimers, IL-12Rβ2-gp130 and IL-12Rβ2-IL-27Rα, and two homodimers, IL-12Rβ2–IL-12Rβ2 and gp130–gp130 [13,14]. Among these subunits, IL-12Rβ2 is mainly expressed by activated natural killer cells and T cells [15,16]. Gp130 is expressed by most immune cells [17], and IL-27Rα is primarily expressed by activated CD8^+^ T cells, CD4^+^ T cells, and monocytes [18]. Once IL-35 binds to the corresponding receptors, signal transduction begins, and signal transducer and activator of transcription (STAT) family members and Janus kinase (JAK) family members are activated. In T cells, its signal transduction involves three receptors, including IL-12Rβ2-gp130, IL-12Rβ2–IL-12Rβ2, and gp130–gp130, and the process is mainly accomplished by activating STAT1 and STAT4 [13]. The two homodimeric receptors can only suppress T cell proliferation, while the IL-12Rβ2-gp130 heterodimeric receptor is able to reduce T cell multiplication and mediate the induction of a potent Treg subset, IL-35-induced regulatory T cells (iTr35) [13]. In Bregs, recombinant IL-35 activates STAT1 and STAT3 pathways by binding with IL-12Rβ2-IL-27Rα, which can produce two Breg subsets that secrete IL-35 (IL-35^+^ Bregs) and IL-10 (IL-10^+^ Bregs) (Table 1) [14,19]. These results show that IL-35 is capable of binding to different receptors in different cell types.

**Table 1:** The expression, signaling pathways, and functions of IL-35

Position	Receptor	STATs	Function
T cells	gp130–gp130	STAT1	Suppressing T cell proliferation
	IL-12Rβ2–IL-12Rβ2	STAT4	Suppressing T cell proliferation
	IL-12Rβ2–gp130	STAT1	Suppressing T cell proliferation
		STAT1, STAT4	Inducing iTr35 generation
B cells	IL-12Rβ2–IL-27Rα	STAT1, STAT3	Promoting conversion of IL-10^+^ Bregs and IL-35^+^ Bregs

## 2 Biological functions of IL-35 in autoimmunity

IL-35 is significant in the progression of inflammation and immune reactions, and IL-35 can mediate immunosuppressive and immunoregulatory functions.

**Figure 1:**
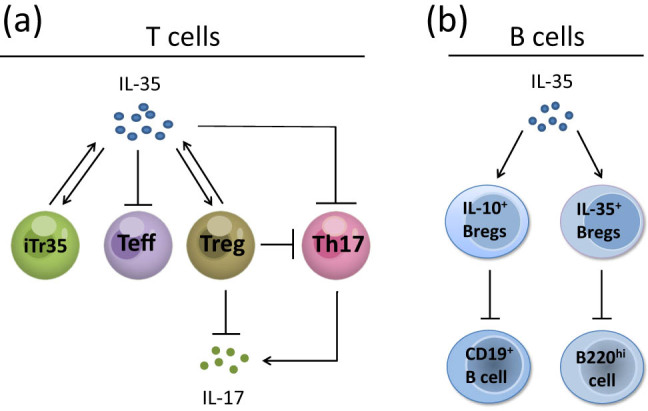
Biological functions of IL-35. (a) IL-35 regulates T cell-mediated immune responses. IL-35 can induce iTr35 generation, and the resulting iTr35 cells produce additional IL-35. IL-35 suppresses Teff proliferation. By upregulating the suppressive function of Tregs, IL-35 can suppress Th17 cell differentiation and IL-17 secretion. (b) IL-35 regulates B cell-mediated immune responses. IL-35 induces IL-10^+^ Bregs and IL-35^+^ Bregs. Moreover, IL-10^+^ Bregs inhibit the propagation of CD19^+^ B cells, and IL-35^+^ Bregs suppress the expansion of B220^hi^ cells. iTr35: IL-35-induced regulatory T cells, Teff: effector T cell, Treg: regulatory T cell, ROR: retinoic acid receptor-related orphan receptor, IL-10^+^ Breg: regulatory B cells secreting IL-10, IL-35^+^ Breg: regulatory B cells secreting IL-35.

In mice, IL-35 expressed by T cells can reduce effector T cell (Teff) multiplication [1]. Furthermore, mouse recombinant IL-35, a functional heterodimer genetically engineered by combining p35 and Ebi3, can strengthen the inhibitory effect of CD8^+^CTLA-4^+^ Tregs on the propagation of autologous T cells [20]. IL-35 inhibits CD8^+^ T cells by influencing cellular immunosuppressive regulation and external regulatory protein stimulation [21]. It has been reported that mesenchymal stem cells, transfected with lentivirus carrying IL-35, stimulate CD4^+^CD25^+^ Tregs proliferation and inhibit CD4^+^ T cells propagation. And in the supernatant of the coculture system containing these three cell subpopulations, the levels of IL-10 and IL-35 increased, while the secretion of IL-17 decreased compared to control groups with non-transfected mesenchymal stem cells or phosphate buffer saline (PBS) [22]. IL-35 inhibits the activation and differentiation of IL-17A^+^ T helper (Th17) cells [23,24]. To investigate the effect of IL-35 on Th17-related transcription factors, a study found that Th17-related transcription factors T-bet and retinoic acid receptor-associated orphan receptor (ROR)γ T were significantly inhibited in mice treated with IL-35 [25]. Another study in PBMC showed that recombinant IL-35 regulates Th17 cell differentiation by inhibiting RORα and RORγ T transcription factors, and inhibits IL-17 mRNA transcription, thereby reducing IL-17 secretion [26]. Inducible costimulator-positive Tregs are able to produce IL-35 to suppress IL-17 production [23]. Tregs are in a position to reduce the propagation of Th17 cells; moreover, IL-35 strengthens the inhibition of Tregs [27]. Additionally, it was observed that IL-35 can maintain the Treg phenotype and inhibit Th17 cell differentiation [24] (Figure 1a). These findings suggest that IL-35 plays an important role in regulating the balance between Tregs and Th17 cells.

As previously mentioned, IL-35 is capable of inducing iTr35 generation, which produces IL-35 in both humans and mice [28]. Induced regulatory T cells are different from thymus-derived natural Treg cells in that they are produced by IL-10 or transforming growth factor-beta (TGF-β) induction [29,30]. However, iTr35 cells mediate immunological suppression only through IL-35, rather than by proven cytokines, including TGF-β and IL-10 [28]. Unlike the currently known TGF-β-induced regulatory T cells, which express Foxp3, iTr35 cells do not express Foxp3 [28,31]. Furthermore, iTr35 cells and IL-35 generate a positive feedback loop in which IL-35 induces iTr35 cell proliferation, and the produced iTr35 cells produce additional IL-35 [28] (Figure 1a).

IL-35 also mediates the immunoregulatory function of Bregs. Recombinant IL-35 induces Bregs and promotes their conversion to IL-10^+^ Bregs and IL-35^+^ Bregs. As mentioned above, this process is accomplished by activating STAT1/STAT3 pathways through the IL-35 receptor, made up of IL-12Rβ2 and IL-27Rα [14,19]. Meanwhile, IL-10^+^ Bregs downregulate the propagation of CD19^+^ B cells, and IL-35^+^ Bregs show a suppressive function of the expansion of B220^hi^ cells [14] (Figure 1b). Tonsil-derived mesenchymal stem cells from humans increase the number of IL-10-producing Bregs through Ebi3, thus reducing the immune response mediated by B cells in mice [32].

IL-35 does not directly affect the viability of human mast cell line cells, but significantly inhibits the viability of human mast cell line cells stimulated by phorbol 12-myristate 13-acetate and A23187 (calcium ionophore) [33]. In addition, IL-35 suppresses histamine release, IL-6 and IL-17 mRNA expression, and mitogen-activated protein kinase (MAPK) phosphorylation in human mast cell line cells stimulated by phorbol 12-myristate 13-acetate and A23187 [33]. All these facts show that IL-35 mediates significant functions in immunological regulation.

## 3 Roles of IL-35 in autoimmune dermatoses

### 3.1 Systemic lupus erythematosus

Systemic lupus erythematosus (SLE) is an autoimmune disease with multiorgan and multisystem involvement, such as skin, kidney, and vascular dysfunctions. The imbalance of CD8^+^ T cells and CD4^+^ T cells in SLE patients results in continued B cell activation to produce various types of autoantibodies and result in the persistence of autoimmunity [34,35,36]. A study found that in Murphy Roths Large (MRL)/lpr mice, a spontaneous lupus-like disease model, the reduction of IL-10^+^ Breg cells and serum IL-10 were accompanied by reduced serum IL-35, prompting that IL-35 may be involved in the regulatory function of Breg cells from lupus [37]. The immune imbalance between Th17 cells and Tregs leads to the destruction of immune homeostasis, which is closely correlated with the development of SLE [38,39,40]. Therefore, it is speculated that IL-35 may help restore the immune balance of SLE patients by limiting the functions of Th17 cells and CD4^+^ T cells and promoting Treg proliferation. Research on juvenile SLE has shown that leukocyte-associated-immunoglobulin-like receptor 1 (LAIR1), one of the observably downregulated differential expression proteins in juvenile SLE patients, binds to the Src homologue 2 domain of protein tyrosine phosphatase non-receptor type 11 (PTPN11) through its two cytoplasmic tyrosine inhibitory motifs, and has a potential inhibitory effect on lymphocytes and leads to dephosphorylation of subsequent kinases. IL-35 leads to the inhibition of the JAK/STAT and MAPK signaling pathways by increasing LAIR1 levels. Then, it adjusts the LAIR1-PTPN11-JAK-STAT-fibronectin 1 interaction network, and ultimately, may mitigate juvenile SLE nephritis [41].

A study by Cai et al. in female MRL/lpr mice showed that in comparison with PBS-treated mice, nephritis and lupus diseases in IL-35-treated mice were obviously remissive [42]. The mRNA levels of Foxp3, IL-35 subunits (p35 and Ebi3), free subunits of IL-35 receptors (IL-12Rβ2 and gp130) from splenic and thymic cells were distinctly elevated in MRL/lpr mice after IL-35 therapy in comparison with PBS-treated MRL/lpr mice [42]. Subsequently, plasma concentrations of gp130 and IL-35 of MRL/lpr mice with mild disease, IL-12Rβ2 and gp130 of MRL/lpr mice with moderate disease, and IL-12Rβ2 of MRL/lpr mice with severe disease after IL-35 therapy were found to be elevated relative to those of PBS-treated MRL/lpr mice [42]. CD4^+^CD25^+^Foxp3^+^ Treg cells were upgraded in the spleen, thymus, and peripheral blood of lupus mice treated with IL-35, so that the CD4^+^CD25^+^Foxp3^+^ Treg/CD4^+^CD25^−^ Teff ratio was obviously upregulated in all groups after IL-35 therapy, and the proportion of IL-10^+^ Bregs obviously increased with IL-35 treatment [42]. The percentage and absolute number of Tregs in the spleen, thymus, and peripheral blood of lupus mice treated with IL-35 overexpression plasmid were higher [41]. Concentrations of pro-inflammatory cytokines in plasma containing IL-17A, IL-6, interferon-gamma (IFN-γ), and tumour necrosis factor-alpha (TNF-α) were significantly decreased, and IL-10, an anti-inflammatory cytokine, was significantly increased in plasma of IL-35-treated mice [42]. It was also shown that SLE-related plasma antibodies (antinuclear antibody and anti-double-stranded DNA antibody) concentrations were significantly reduced, demonstrating that IL-35 probably mediates the suppression of SLE through the immunoregulatory functions of Tregs and Bregs [42]. A separated peripheral blood mononuclear cell (PBMC) culture experiment revealed lower IL-35 levels in active SLE patients relative to healthy controls (HCs) [43]. IL-35 levels in the serum of active SLE patients were lower than those of non-active SLE patients, and a negative relationship between SLE Disease Activity Index (SLEDAI)-2k scores and serum IL-35 levels was observed [44,45]. The proportion of CD4^+^ T cells expressing EBI3 in peripheral blood of patients with active SLE was lower than that of HCs and inactive SLE patients and was negatively correlated with the SLEDAI score [45]. These results may indicate that IL-35 and Ebi3-expressing CD4^+^ T cells may play a protective part in the development of SLE, and their IL-35 levels may be used as a measure of SLE activity. IL-35 levels in the serum of SLE patients with lupus nephritis were obviously lower than those of SLE patients without lupus nephritis, and IL-35 might be a possible biomarker for kidney damage related to SLE [44]. IL-35 levels in the plasma of newly diagnosed SLE patients were significantly decreased compared with HCs [46]. The decrease of IL-35 quantity in patients with newly diagnosed SLE probably occurs since the plasma concentrations of IL-35 and the number of circulating IL-35^+^ Bregs are both decreased [46]. The expression levels of gp130 on CD4^+^ helper T (Th) cell surface of patients with severe SLE were lower and were negatively related to SLEDAI, and the elevation of soluble forms of gp130 may be related to the reduced expression of gp130 on CD4^+^ T cell surface in patients with SLE [47]. The amounts of CD4^+^CD25^+^ Tregs were in smaller quantities in moderate and severe SLE patients in comparison with HCs [47]. Data in this research revealed that the proliferation of Tregs is related to the gp130 expression level on the surface of CD4^+^ Th cells, which may suggest that the decreased gp130 expression on CD4^+^ Th cells was associated with the downregulated population of Tregs [47].

However, contrary to the studies mentioned above, some researchers reported that IL-35 levels in serum of active SLE patients were higher than those in HCs [48,49]. Qiu et al. observed that higher levels of IL-35 in serum of active SLE patients were reduced after treatment with large doses of prednisone [48]. Therefore, we hypothesized that the reason for the difference in the serum IL-35 concentration compared with the aforementioned studies might partly be related to the fact that active SLE patients in those studies had been treated with glucocorticoids. IL-35 levels in serum were also found obviously increased in newly diagnosed SLE patients in comparison with HCs [50]. In another study, soluble gp130 and IL-35 levels in plasma were increased in newly diagnosed severe SLE patients in comparison with HCs, and Ebi3 and p35 mRNA levels in PBMCs of severe SLE patients were also increased [47]. It is shown that the mRNA level of Ebi3 and p35 in B cells had a considerable increasing trend in the SLE patients compared with HCs [50]. A study that examined subsets of CD3^+^CD4^+^, CD3^+^CD4^−^, and CD3^−^CD4^−^ lymphocytes in patients with SLE found no significant differences in the levels of IL-12Rβ2 and gp130 on the surface of the subsets studied [49]. However, the study did not distinguish between living and dead cells, nor did it distinguish Treg independently. The possible reasons for these different results may be that different samples, including PBMCs, plasma, and serum, were tested, different kits were used, and only Chinese patients were studied [51]. Therefore, future experiments with larger sample size and experiments in other ethnic groups are needed for verification.

### 3.2 Roles of IL-35 in psoriasis

Psoriasis is a chronic disease featuring inflammation. Its characteristics include abnormal infiltration, activation of T cells, and excessive propagation of keratinocytes. The IL-23/IL-17 axis and Th17 cells are both significant in the pathological process of psoriasis [52,53,54]. The IL-35 levels in the serum of psoriasis vulgaris patients before treatment were prominently lower than those in HCs, but the IL-35 levels in serum were obviously upregulated with routine treatment [55]. Plasma IL-35 levels were lower in psoriasis patients in comparison with HCs [56,57]. The level of IL-35 in psoriatic skin biopsies was lower than that in the surrounding skin and normal controls [58]. The study of plaque psoriasis found that the skin lesion of plaque psoriasis patients gradually recovered after treatment with adalimumab; however, the plasma IL-35 level was lower in the adalimumab group [57]. The possible reason for the contrary result is that the observation time is short and the skin lesions in patients with adalimumab treatment are more severe. Expression levels of plasma IL-35 were negatively correlated with IL-17, IL-22, IL-23, TNF-α, and IFN-γ [56]. Meanwhile, its expression was positively related to the levels of IL-10 and TGF-β in psoriasis patients [56]. It is suggested that IL-35 may influence the pathogenesis of psoriasis by regulating the generation of cytokines related to Th17 cells or Tregs.

Similar results were seen in keratin 14-vascular endothelial growth factor A (VEGF-A)-transgenic mouse models. Compared with the control group, which was transfected with the pcDNA3.1 vector, the IL-6 and IL-17A levels in ear tissues and serum supernatant were lower, there were fewer IL-17-secreting CD4^+^ T cells of lymph nodes and spleen, and the number of CD4^+^ T cells that secrete IL-10 in lymph nodes and spleen were increased in mice that were transfected with the plasmid coding human IL-35 (pIL-35) group [59]. The levels of classically inflammatory macrophages (M1) in ear tissues and spleens of the pIL-35 group were decreased, while the level of alternatively anti-inflammatory macrophages (M2) was increased, and the M1/M2 ratio was decreased. M1 and M2 are considered to be linked with the inflammatory response in wound healing [60]. IL-35 induced immunosuppression in keratin 14-VEGF-A-transgenic mice via the reduction of the local infiltration of macrophages and the decrease in the M1/M2 proportion [59]. The treatment of IL-35 improved the redness, scaliness, severe ear thickening and swelling, and other psoriatic lesions in psoriasis mice models [59,61]. Rather, it has been reported that no IL-35 was present in the serum in an experiment conducted in Brazilian psoriasis patients [62]. IL-35 may be a potential target to monitor or treat psoriasis, but more studies are needed to confirm its effects in psoriasis.

### 3.3 Systemic sclerosis

Systemic sclerosis (SSc), an autoimmune connective tissue disease, develops due to abnormal activation of fibroblasts, which produce excessive collagen, resulting in skin and internal visceral fibrosis.

Macrophages, T cells, B cells, and other immune cells are implicated in SSc [63]. The role of IL-35 in the pathogenesis of SSc is unclear. Results of a study on SSC showed that IL-35 was elevated in patients compared to controls, and IL-35 inhibited CD4^+^ T lymphocyte proliferation and induced Treg differentiation [64]. IL-35 can inhibit type I collagen expression in normal fibroblasts and SSc dermal fibroblasts, and reduce the stability of collagen mRNA in normal fibroblasts [65]. These effects of IL-35 were realized by subunit Ebi3, while another subunit p35 was found to have no such effect in the study [65]. Skin injection of Ebi3 improved skin fibrosis in SSc mice models, further suggesting that IL-35 may have potential therapeutic effects to improve fibrosis during the pathogenesis of SSc [65].

However, other studies suggested that IL-35 promotes collagen production during the pathogenesis of SSc. According to the injury level of the skin, SSc is classified as either diffuse cutaneous SSc or limited cutaneous SSc [66]. These two kinds of SSc patients had higher IL-35 concentrations in serum than that in HCs, while no differences were found in serum IL-35 production between the two types of patients, and patients with lung fibrosis had higher IL-35 levels relative to those without fibrosis [67]. In another study, there was significant upregulation of p35, Ebi3, and IL-35 in lesioned skin from SSc patients in comparison with HCs at both the protein and the mRNA levels [68]. IL-35 expresses and releases increasingly under stimulation of TGF-β, and IL-35 can induce resting fibroblasts to differentiate into myofibroblasts, thereby increasing the release of collagen protein [68]. It has also been observed that IL-35 levels in the serum of SSc patients had a negative relationship with disease duration, and the results of capillary microscopy showed that early SSc patients have higher serum IL-35 levels in comparison with active or late SSc patients [68]. Likewise, Tang et al. observed that the IL-35 levels in the serum of SSc patients were obviously higher than those of the HCs, but after 3 months of treatment, the IL-35 levels in the serum of SSc patients were reduced primarily [69]. It can be inferred that the elevation of serum IL-35 was correlated with the early skin and pulmonary fibrosis of SSc, results that are opposite to the anti-inflammatory effects of IL-35 in other mentioned diseases. Yayla et al. also discovered higher concentrations of IL-35 in SSc patients’ serum, but they found that it was negatively related to C-reactive protein (CRP), Medsger disease severity score, and modified Rodnan skin score [70]. However, IL-35 levels were not different between the SSc patients with or without lung fibrosis in this study; therefore, more studies need to be performed to verify these results. In a word, IL-35 might be one of the serologic biomarkers indicating the inflammatory status of SSc.

### 3.4 Dermatomyositis

Dermatomyositis (DM) is an autoimmune disease involving the skin and striated muscle. There are different lymphocyte subsets that accumulate in different regions of the muscle in DM patients [71]. The membrane attack complex is composed of B cells and C5-9 complement bodies. T cells, macrophages, dendritic cells, and B cells are all possibly relevant to the pathogenesis of DM [71]. However, there are still few studies exploring the relationship between DM and IL-35. It has been observed that serum concentrations of IL-35 of DM patients are overexpressed relative to HCs [72,73,74]. Surprisingly, the results of the research revealed that IL-35 levels in serum were not correlated to peripheral blood lymphocyte subgroup counts in idiopathic inflammatory myopathy patients [72]. The results of previous experiments showed that the populations of Tregs in the peripheral blood of DM patients were decreased in comparison to HCs [75,76]. There were also decreases in populations of CD3^+^ cells, CD3^+^CD8^+^ cells, and CD3^+^CD4^+^ cells in active DM patients compared with patients with inactive DM and HCs [77]. These might mean that the source of IL-35 may come from non-T cell sources but not peripheral blood T cells.

Higher serum expression of IL-35 was found in active DM patients than in remissive individuals [73]. It is observed that serum levels of IL-35 were the highest in DM patients whose disease durations were shorter than 6 months, while the lowest serum frequency of IL-35 was in DM patients with disease duration longer than 12 months compared with HCs. Moreover, the IL-35 frequency in the serum of untreated patients was also higher than in those who relapsed [73]. Additionally, the serum concentrations of IL-35 in idiopathic inflammatory myopathy patients were negatively correlated with the disease course [72,74]. Serum IL-35 quantity had a positive relationship with erythrocyte sedimentation rate (ESR), CRP, lactate dehydrogenase (LDH), creatine kinase, and visual analogue scale [73]. As these measures are correlated with disease activity, and creatine kinase and LDH are the indicators to estimate muscle damage, therefore, serum IL-35 may be a potential biomarker to assess disease activity or muscle damage of DM. Exogenous human recombinant IL-35 downregulated IL-17 and TNF-α production in PBMCs stimulated by lipopolysaccharide from DM patients compared with HCs [73]. These findings suggest that IL-35 may have the effect of immune suppression on DM. However, the possibility that elevated IL-35 probably mediates a pro-inflammatory role in DM patients cannot yet be ruled out. The function of IL-35 in DM pathogenesis still needs to be explored in the future. The experimental results of the above-mentioned diseases in each article are summarized in Table 2.

**Table 2:** IL-35 levels and biological functions in autoimmune dermatoses

Disease	Source	Control group	Sample	Findings	Function	Ref.
SLE	MRL/lpr mice	PBS-treated MRL/lpr mice	Peripheral blood, splenic, and thymic cells	Tregs increased in IL-35-treated MRL/lpr mice	Suppress inflammation	[41]
			—	Nephritis and lupus diseases in IL-35-treated MRL/lpr mice were remissive	Suppress inflammation	[42]
			Splenic and thymic cells	Foxp3, p35, Ebi3, and free gp130 and IL-12Rβ2 increased in IL-35-treated MRL/lpr mice		[42]
			Plasma	IL-35, gp130, and IL-12Rβ2 increased in IL-35-treated MRL/lpr mice		[42]
			Peripheral blood, splenic, and thymic cells	CD4^+^CD25^+^Foxp3^+^ Tregs and IL-10^+^ Bregs increased in IL-35-treated MRL/lpr mice		[42]
			Plasma	IFN-γ, TNF-α, IL-6, and IL-17A decreased, and IL-10 increased in IL-35-treated MRL/lpr mice		[42]
			Plasma	Antinuclear antibody and anti-double-stranded DNA antibody decreased in IL-35-treated MRL/lpr mice		[42]
	Human	Healthy individuals	PBMC supernatants	IL-35 decreased in active SLE patients	Suppress inflammation	[43]
			Plasma	IL-35 decreased in newly diagnosed SLE patients	Suppress inflammation	[46]
			PBMCs	IL-35^+^ B cells decreased in newly diagnosed SLE patients		[46]
		Inactive SLE patients	Serum	IL-35 decreased in active SLE patients	Suppress inflammation	[44,45]
			Serum	IL-35 levels were negatively correlated with SLEDAI-2 k		[44,45]
		SLE patients without lupus nephritis	Serum	IL-35 decreased in SLE patients with lupus nephritis		[44]
		Healthy individuals	Serum	IL-35 increased in active SLE patients	Promote inflammation	[48,49]
			Serum	IL-35 increased in newly diagnosed SLE patients	Promote inflammation	[50]
			B cells	P35 and Ebi3 increased in SLE patients		[50]
			Plasma	IL-35 and soluble gp130 increased in severe SLE patients	Promote inflammation	[47]
			PBMCs	P53 and Ebi3 increased in severe SLE patients		[47]
			Th cells	Gp130 decreased in severe SLE patients		[47]
			Th cells	Gp130 levels were negatively correlated with SLEDAI		[47]
			PBMCs	CD4^+^CD25^+^ Tregs decreased in severe and moderate SLE patients		[47]
Psoriasis	Human	Healthy individuals	Serum	IL-35 decreased in patients with psoriasis vulgaris	Suppress inflammation	[55,56]
			Plasma	IL-35 decreased in psoriasis patients	Suppress inflammation	[56,57]
			Plasma	IL-35 levels were negatively correlated with IFN-γ, TNF-α, IL-23, IL-17, IL-22, and positively correlated with TGF-β and IL-10	Suppress inflammation	[56]
			Skin	IL-35 decreased in psoriatic skin biopsies	Suppress inflammation	[58]
	Keratin 14-VEGF-A-transgenic mice	Mice transfected with pcDNA3.1 vector	Serum and ear tissues supernatant	IL-6 and IL-17A decreased in mice transfected with pIL-35	Suppress inflammation	[59]
			Spleen and lymph node	IL-17 secreting CD4^+^ T cells decreased and IL-10 secreting CD4^+^ T cells increased in mice transfected with pIL-35		[59]
			Spleen and ear tissues	M1 decreased and M2 increased in mice transfected with pIL-35		[59]
			—	Psoriatic lesions were improved in mice transfected with pIL-35		[59]
SSc	Human	Healthy individuals	Serum	IL-35 increased in SSc patients, inhibited CD4^+^ T lymphocyte proliferation, and induced Treg differentiation	Suppress inflammation	[64]
			Serum	IL-35 increased in SSc patients	Promote inflammation	[67,68,69,70]
			Serum	IL-35 levels were negatively correlated with disease duration, modified Rodnan skin score, Medsger disease severity score, and CRP		[68,70]
			Skin	IL-35 increased in SSc patients, and upregulated differentiation of myofibroblasts		[68]
		Fibroblasts without IL-35	—	Type I collagen expression were inhibited by IL-35 in normal fibroblasts and SSc dermal fibroblasts	Suppress inflammation	[65]
	SSc mice model	Balb/c mice injected with PBS	Skin	Skin fibrosis was improved in mice skin injected with Ebi3	Suppress inflammation	[65]
DM	Human	Healthy individuals	Serum	IL-35 increased in DM patients	Suppress inflammation	[72,73,74]
			Serum	IL-35 levels were negatively correlated with disease duration		[72,74]
			Serum	IL-35 levels were positively correlated with ESR, CRP, visual analogue scale, creatine kinase, and LDH		[73]
		Recurrent patients	Serum	IL-35 increased in untreated DM patients		[73]
		Patients in remission	Serum	IL-35 increased in active DM patients		[73]
		Healthy individuals	PBMCs	Human recombinant IL-35 inhibited IL-17 and TNF-α productions of PBMCs stimulated by lipopolysaccharide from DM patients		[73]

SLE – systemic lupus erythematosus, SSc – systemic sclerosis, PBMC – peripheral blood mononuclear cell, SLEDAI-2k – systemic lupus erythematosus disease activity index-2k, pIL-35 – plasmid coding human IL-35 sequence, CRP – C-reactive protein, DM – dermatomyositis, ESR – erythrocyte sedimentation rate, LDH – lactate dehydrogenase.

## 4 Conclusions

IL-35 acts as a significant inhibitory cytokine in the immunity system, modulating dysfunctional B cells and T cells and regulating various immune-related inflammatory factors. For this reason, IL-35 is important in autoimmune dermatosis. In SLE and psoriasis, a large amount of evidence supports a protective immunosuppressive effect of IL-35; however, it may promote fibrosis, which may suggest the development of inflammation in SSc. IL-35 might play an immunosuppressive role in DM, but this hypothesis remains to be verified. Therefore, its role in immune regulation may vary greatly in different autoimmune diseases, and more studies are needed in the future to elucidate its function of the occurrence and development of different diseases. Although IL-35 plays different roles in the immune regulation of different diseases, it can still be regarded as a treatment target for autoimmune diseases. If IL-35 expression is decreased in a disease, recombinant IL-35 can be used for treatment. If IL-35 expression is significantly upregulated in some diseases, treatment can be performed by reducing the expression of IL-35. With further research on IL-35 in the future, IL-35 is expected to be a new therapeutic target for autoimmune diseases.

## Abbreviation list

Breg: regulatory B cell
CRP: C-reactive protein
DM: dermatomyositis
Ebi3: Epstein-Barr virus-induced gene 3
ESR: erythrocyte sedimentation rate
HC: healthy control
IFN-γ: interferon-gamma
IL-10^+^Breg: regulatory B cells secreting IL-10
IL-35^+^Breg: regulatory B cells secreting IL-35
IL-35: interleukin-35
iTr35: IL-35-induced regulatory T cells
JAK: Janus kinase
LAIR1: leukocyte-associated-immunoglobulin-like receptor 1
LDH: lactate dehydrogenase
M1: inflammatory macrophages
M2: anti-inflammatory macrophages
MAPK: mitogen-activated protein kinase
MRL: Murphy Roths Large
PBMC: separated peripheral blood mononuclear cell
PBS: phosphate buffer saline
pIL-35: plasmid coding human IL-35
PTPN11: protein tyrosine phosphatase non-receptor type 11
ROR: receptor-related orphan receptor
SLE: systemic lupus erythematosus
SLEDAI: systemic lupus erythematosus disease activity index
SSc: systemic sclerosis
STAT: signal transducer and activator of transcription
Teff: effector T cell
TGF-β: transforming growth factor beta
Th cell : T helper cell
TNF-α: tumour necrosis factor-alpha
Treg: regulatory T cell
VEGF: vascular endothelial growth factor
